# Imaginary Diseases

**Published:** 1879-08

**Authors:** E. H. Gibbs


### IMAGINARY DISEASES.

By E. H. Gibbs, M. D.

The actual cares of life are sufficient for most people without the additional burden of *imaginary* evils. In a former number of the Journal of Health I referred to the well-known fact that of all those who die suddenly of supposed "heart disease," not one in twenty has *any disease whatever* of that organ.

A great majority of these deaths are caused by a sudden congestion of the brain or lungs. There are thousands of heart-sound people who are dragging out miserable lives in the constant fear of sudden death from this trouble. I referred to a case I had been called to see some months before that article was published. The attending physician said it was a case of "hypertrophy" or enlargement of the heart *in its last stage.* The man is by trade a house-painter, but he had done no work during the previous six months, and for four weeks had not left his room. I saw at a glance that there were no visible signs of heart trouble, and a most searching examination of the chest failed to reveal any. I soon became satisfied that it was a case of "dyspepsia," aggravated, doubtless, by lead poisoning and the obstinate condition of constipation from which he suffered. I ventured to assure him that the heart was sound and that the frequent and violent palpitations which were supposed to be conclusive evidence of heart disease were due to other causes, which I carefully explained to him. Treatment was directed to the relief of the actual trouble, and within a week the man was attending to his business. I see him occasionally, and he says that beyond an occasional but slight palpitation—that sometimes seizes but no more alarms him—he enjoys excellent health.

The treatment in these cases is to abstain as far as possible from the use of drugs and employ in their stead wholesome food, exercise, and fresh air.

"Nelaton's Suppository" is not a drug, but simply a local application. Its value was manifested in this case, since it proved to be the only remedy that had power to relieve the constipation, which was the greatest obstacle to recovery. A similar case, and one of far more interest to me, has recently come under my observation: A gentleman called and requested that I would treat him for "Bright's Disease" of the kidneys. He said the ordinary tests for albumen had been made by competent persons a number of times, and this evidence of "Bright's Disease" found in every instance. I made the ordinary test and discovered, as I supposed, considerable albumen. Still there were certain symptoms, some of them, it is true, very obscure, that I had invariably observed in all cases of genuine "albuminuria," that seemed to be wanting here. My patient was nervous and despondent, and perhaps his confidence in my skill was lessened by my doubts in regard to what he believed to be a foregone conclusion.

His mental condition was bad enough, but the supposed disease was not active; in truth there was marked improvement in the course of a few days under the treatment I had prescribed. Then a most careful examination of the urine was made, when it was found that there was the appearance of coagulation, as if it had been albumen, but, on careful examination, the product proved to be only a sediment resulting from slight malarial congestion of the liver. When this favorable condition was made known to my patient he soon recovered his spirits, and after a continuation of the treatment for about ten days longer recovered his health also; and there is not probably a sounder man in New York to-day than he. Suppose the first examinations that were made had been regarded as conclusive—which the patient himself seemed determined they should be—what would have been the result?

"Bright's Disease" is generally supposed to be incurable—though this is a great mistake, as most cases recover with proper treatment—this man believed it fatal. He would have brooded over his imaginary ailment until he had so weakened the vital forces as to invite the disease, and he would probably in time have died of it. If the number of victims who are dying by similar processes could be known we should be horrified at the long list of these unfortunates.

There is another and a very numerous class of young persons to whom I will give a word of advice. Through ignorance or recklessness many acquire habits that are destructive of health to the exact extent of their indulgence. The end is despondency, lunacy, and an early grave.

The best thing is to go in confidence to your family physician and state the case fully and truthfully, and then follow his advice to the letter. He will neither betray your confidence nor think evil of you In fact, he will do his duty as a physician, and think no more about it. Above all things pay no attention to the attractive advertisements of so-called *physicians* who profess to be able to cure you. As a class they are the most shameless pack of ruffians to be found. They are utterly ignorant of everything that pertains to medical science, and they live by fleecing the young and ignorant, whose fears they work upon.

